# Simple Plex^™^: A Novel Multi‐Analyte, Automated Microfluidic Immunoassay Platform for the Detection of Human and Mouse Cytokines and Chemokines

**DOI:** 10.1111/aji.12512

**Published:** 2016-05-11

**Authors:** Paulomi Aldo, Gregory Marusov, Danielle Svancara, James David, Gil Mor

**Affiliations:** ^1^ Division of Reproductive SciencesDepartment of ObstetricsGynecology and Reproductive SciencesYale University School of Medicine. New HavenCTUSA; ^2^ ProteinSimpleSan JoseCAUSA; ^3^ R&D SystemsMinneapolisMNUSA

**Keywords:** Chemokines, cytokines, immunoassay, multiplex, Simple Plex

## Abstract

**Problem:**

Quantitative measurement of proteins in bodily fluids or cellular preparations is critical for the evaluation of biomarkers or the study of complex cellular processes. While immunoassays are the most common quantitative approach used so far, they are not practical for the evaluation of multiple proteins. Microfluidic technology allows a fine spatial control in immobilizing proteins and biomolecules inside microchannels, eliminating cross‐reactivity between competing analytes, and allowing rapid and sensitive detection of targeted antigens for multiple applications. We report the characterization and validation of the Simple Plex^™^ platform for the detection and quantification of cytokines and chemokines from human and mouse samples.

**Method:**

Cytokine and chemokine expression levels were determined using Simple Plex cartridges from ProteinSimple. Serum samples were obtained from the Yale Biorepository.

**Results:**

Our data demonstrate an excellent correlation between the results obtained with Simple Plex and conventional immunoassays such as ELISA and Luminex.

**Conclusion:**

We describe the characterization and validation of Simple Plex, a novel multi‐analyte, automated microfluidic platform that allows the evaluation of cytokines and chemokines from human and mice biological samples. Simple Plex showed significant advantages over traditional approaches in terms of low sample volume requirements, sensitivity and dynamic range, coefficient of variation, and reproducibility.

## Introduction

Quantitative measurement of several proteins in bodily fluids or cellular preparations is critical for the evaluation of biomarker candidates or the study of complex cellular processes, such as inflammation, cancer, and autoimmune diseases.^1^, ^2^, ^3^ Immunoassays are the most common quantitative approach used and have been extensively characterized in terms of sensitivity, reproducibility, and variability.^4^, ^5^ However, due to labor intensity existing immunoassays are not practical for the evaluation of multiple proteins, nor are they cost‐effective.^4^ Consequently, multiplexing capability is becoming a critical parameter of biomarker validation and testing as biomarkers are increasingly utilized in all facets of life science research and clinical diagnostics. The adoption of multiplexed testing formats in either clinical research or diagnostics has been severely limited for many reasons, including technical concerns regarding assay reproducibility, cross‐reactivity between the disparate antibody assay components, decreased sensitivity, increased variability, and reported non‐correlation of results with existing methods, including single‐parametric ELISAs.^6^, ^7^ As such, many potential multiplex tests continue to be performed as single‐test ELISAs in a parallel processing format.

Using design strategies developed by the semiconductor industry, microfluidic integration offers a rapid ‘sample‐in, answer‐out’ capability.^8^, ^9^ Microfluidic technology allows a fine spatial control in immobilizing proteins and biomolecules within microchannels, allowing multiplexing and multiparameter assays.^10^ The overall design of self‐contained microfluidic devices and automation reduces human sampling or pipetting errors. Furthermore, analytic immunoassays in microfluidic formats allow a rapid and sensitive detection of one or several targeted antigens for multiple applications including clinical diagnostics, protein sensors, and environmental analysis.^11^

ProteinSimple has developed a novel automated immunoassay platform, Simple Plex^™^, for the rapid and sensitive detection of up to four targeted protein antigens across multiple biological sources. Simple Plex^™^ is an integrated immunoassay system that consists of a disposable microfluidic cartridge and an automated analyzer, the Ella instrument. The system enables simultaneous quantitation of four analytes from sixteen individual samples, or a single analyte from 72 samples, in a single disposable microfluidic cartridge, within an hour. Each sample is analyzed in a unique parallel channel within the cartridge. Simple Plex^™^ assays overcome many limitations of traditional plate‐based ELISAS such as large sample volume requirements, slow reaction rate, intensive labor requirements, and cost inefficiencies, especially when analyzing multiple analytes.

The objective of this study was to characterize and validate the Simple Plex^™^ platform for the detection and quantification of cytokines and chemokines from human and mouse samples. We demonstrate the sensitivity and specificity of the system for the quantification of analytes in multiple biological sources, including human tissue and cell lysates and supernatants, human serum and plasma, as well as mice serum and tissue lysates. The system is highly reproducible, fully automated, easy to use and economically efficient.

## Materials and methods

### Simple Plex Assay Protocol

Simple Plex tests are highly automated and require no manual washes, ensuring ease of operation. The test is performed by selecting a cartridge with the desired analyte panel, adding a minimum of 50 μL of sample reagent (diluted biological sample, quality control, or calibration point) into each sample inlet, and adding a minimum of 350 μL of provided wash buffer into corresponding inlets. The user initiates the run via software designed to facilitate proper data organization and execution of automated scripts without user intervention. The entire test process is enabled by powerful and intuitive Simple Plex Runner software that guides the user.

All immunoassay operations (including prime system, flow samples and split them into channels, sample incubation, wash, rehydrate and flow secondary antibody, wash, rehydrate and flow streptavidin dye conjugate, incubate, wash, scan) are processed automatically. About an hour after test initiation, results (triplicate results for every analyte of each sample) are showed. Data review is accomplished via the ‘Results’ tab on the software. Raw signal levels (relative fluorescence units, RFUs), mean signal values, standard deviation, and coefficient of variance (CV) for each glass nanoReactor (GNR) value are provided. RFU values are automatically backfit to produce an analyte concentration per analyte/sample using the calibration methodology detailed below. An additional layer of data management software, Simple Plex Explorer, is provided for further analysis. Data can also be easily exported to an Excel format, and a 21 CFR Part 11 enabling software format is also available.

### Calibration Methodology

A key aspect of a quantitative immunoassay is its process of quantification. Typically, most immunoassays rely on a calibration process wherein a known calibrator series is run alongside unknown samples, to generate a calibration curve. The raw signals from these calibrators are correlated with known concentration values and the correlation is transformed into a mathematical function, which forms the basis for converting raw data from unknown samples into a concentration value (pg/mL).

For existing immunoassay technologies, the process of creating a calibration curve for every target analyte is essential, due to inherent assay variability, largely resulting from technical inadequacies and extensive manual intervention. The use of a calibration curve alongside every test somewhat mitigates these issues; however, it reduces sampling efficiency and increases assay costs. Ella's ‘hands‐free’ features requiring no manual washes, coupled with its technological innovations, allow factory calibration of each Simple Plex assay. Each test cartridge contains calibration‐related parameters affixed within the cartridge barcode, making it unnecessary to run a calibration curve alongside test samples.

### Validation of Simple Plex versus Quantikine ELISA

Human IL‐1b, IL‐6, IL‐10, and TNF‐a Quantikine kits were obtained from R&D Systems (Minneapolis, MN, USA). Assays were performed at R&D Systems in Minneapolis according to the manufacturer's instructions. Thirty‐two human plasma and serum samples per analyte were measured by Quantikine, and identical frozen aliquots were provided for Simple Plex analysis. To cover the entire dynamic range of both assay platforms, in certain cases serum or plasma samples were spiked with human cell supernatant containing elevated levels of endogenous analytes. After Simple Plex analysis of the same samples, Quantikine‐measured values for each sample were provided for correlation analysis.

### Validation of Simple Plex versus Luminex

Human Bio‐Plex Pro IL‐6, IP‐10, TNF‐α, and IL‐1β assays were purchased from Bio‐Rad Laboratories (Hercules, CA, USA). Assays were performed according to the manufacturer's instructions as previously described.^12^, ^13^, ^14^ Cartridges containing IL‐6, IP‐10, TNF‐α, and IL‐1β were run alongside the Luminex plates, and data were converted from relative units/RFU to concentration (pg/ml) to directly compare formats.

### Biological Samples

Cells lysate and supernatant samples were collected from human CD14 + peripheral blood monocytes (HIC protocol# 9903010425) treated with or without 10 ng/mL LPS (Escherichia coli 055:B5; Sigma‐Aldrich, St. Louis, MO, USA) for 24 hr. Serum samples were collected from individuals as part of the Reproductive Sciences Biorepository (HIC protocol #1006007034). Tissue lysate and supernatant from C57bl/6 mice spleen and peritoneal macrophages (IACUC protocol#2013‐11132) were prepared as previously described.^13^

### Statistical Analysis

Simple Plex cartridges were run according to the manufacturer's instructions, and data were processed automatically using default software settings as outlined in the Ella User's Guide. Briefly, sample concentration values in pg/mL are automatically calculated by fitting relative fluorescence units (RFUs) to calibration curve parameters provided with each cartridge and are adjusted for user‐defined dilution factors. Curve parameters for each cartridge are calculated by fitting a 5‐parameter sigmoid curve to factory‐generated calibration curve data using a Levenberg–Marquardt algorithm. Assay performance characteristics (LODs, LOQs, etc.) are calculated as indicated in Table 1 legend according to the FDA guidance (Analytical Procedures and Methods Validation) (37 2000; Bioanalytical Method Validation (2001).

**Table 1:** LODs and LOQs for Curves in Fig. 4

Analyte	Species	LOD (RFU)	LOD (pg/mL)	LLOQ (pg/mL)	ULOQ (pg/mL)
IL‐1b	Human	1.23	0.33	1.60	1000
IL‐6	Human	1.25	0.34	1.60	5000
IL‐10	Human	0.19	0.32	1.60	5000
TNFa	Human	1.05	0.49	1.60	5000
IL‐17A	Human	1.48	2.73	1.31	2000
CCL3	Human	0.94	0.42	3.28	2000
OPN	Human	1.17	7.29	26.21	6400
CXCL10 (IP10)	Human	0.77	0.98	8.19	5000
CCL2 (MCP‐1)	Mouse	0.21	0.13	0.21	2000
CXCL2	Mouse	0.15	0.07	0.52	2000
IL‐6	Mouse	0.33	0.18	0.52	5000
TNF‐a	Mouse	2.37	1.11	1.31	5000

LOD (RFU) = 0 pg/ml mean RFU + 3× StdDevs; LOD (pg/mL) = LOD (RFU) backfit to Factory Calibration (FC) curve; LLOQ (pg/mL) = lowest FC curve pt where CV <20% and % recovery backfit to FC = 80–120%; ULOQ (pg/mL) = lowest FC curve pt where CV <20% and % recovery backfit to FC = 80–120%.

For linear correlation analyses, samples were analyzed in identical fashion at two different locations or on two different immunoassay platforms. Measured concentrations at each site or on each platform were plotted against each other in Excel, and linear regression analysis using a y‐intercept of zero was used to calculate *R* ^2^ values.

## Results and discussion

### Simple Plex ^™^ Immunoassay Components

**Figure 1:**
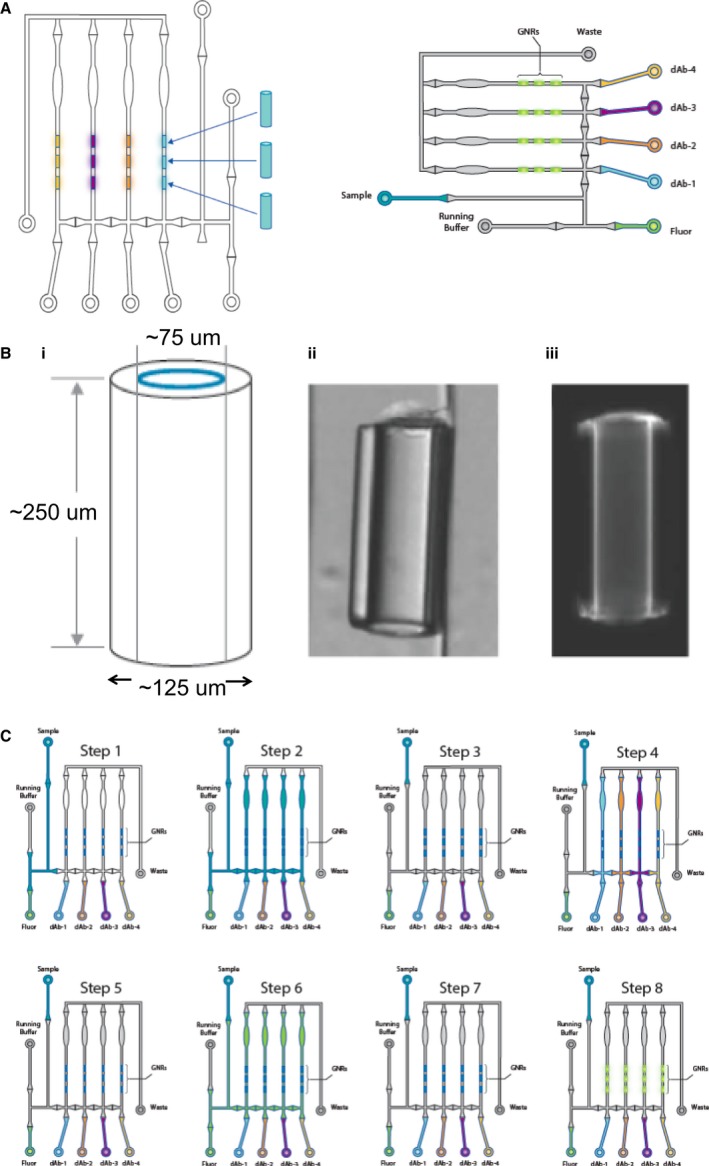
(A) Ella splits each sample across four parallel, isolated microfluidic channels. Each channel has a single‐plex immunoassay for a specific analyte, therefore avoiding antibodies' cross‐reactivity. (B) Structure of the glass nanoreactors (GNRs). GNRs are miniature hollow chambers composed of glass capillaries 250 ± 25 μm (length) by 125 ± 6 μm (outer diameter) with an inner diameter of 75 ± 3 μm (i). Three GNRs are present in each analyte‐specific channel in every circuit. (ii) and (iii) are representative GNRs viewed in light field and fluorescence imaging. C. The Simple Plex assay work flow shown for one of the sixteen circuits: Step 1: The system is primed with sample. Step 2: Sample is pumped through the circuit and split evenly between the four channels containing analyte‐specific GNRs. Step 3: After sample incubation, the circuit is cleaned with wash buffer. Step 4: Analyte‐specific Abs are individually pumped into their respective channels to bind analyte capture on the GNRs. Step 5: Unbound Abs are removed with wash buffer. Step 6: Detect fluor is flowed into all channels, binding bound Abs. Step 7: Residual detect fluor is removed with wash buffer. Step 8: Detect fluor is excited with 631 nm laser, and the signal is read with a CCD camera.

Simple Plex immunoassay system has two components: (i) a microfluidic analyzer, Ella (Fig. S1), and (ii) an assay kit that contains a disposable cartridge, and all the reagents are required to run the test (Simple Plex) (Fig. S2). The desktop Ella Analyzer (21 inches × 18 inches × 14 inches, weight 20 lbs.) is connected to a PC computer via USB and Ethernet cables. The PC has the Simple Plex Runner instrument control software, the Simple Plex Explorer data analysis software, and a barcode scanner to streamline data entry. The Simple Plex microfluidic cartridge utilizes two innovative features: (i) each test sample is actively drawn and split into discrete parallel channels by a combination of micropumps and valves, and (ii) these channels each contain 3 biological substrates, the (GNRs)(Fig. 1A). GNRs are miniature hollow chambers composed of a glass capillary 250 ± 25 μm (length) by 125 ± 6 μm (outer diameter) with an inner diameter of 75 ± 3 μm (Fig. 1B). Each GNR functions as a reaction vessel for antigen–antibody interactions; their chemical and optical properties are ideal for the attachment and detection of antibodies and other biomolecules. Every Simple Plex cartridge is affixed with a barcode that contains the associated factory‐stored calibration curves. Simple Plex cartridges are self‐contained; all reagents, including capture antibodies, biotinylated detection antibodies, and streptavidin–dye conjugate, reside in appropriate locations within the cartridge. The cartridge also contains a reservoir for waste collection. The unique construction of Simple Plex cartridges results in a multi‐analyte, not multiplex, format for assays (Fig. 1A). The setup of the cartridge allows for each sample to be run in triplicate for each analyte and prevents any interaction between the antibody components for each biomarker, thereby eliminating potential cross‐reactivity between antibodies as observed in other biomarker platforms such as Luminex (Fig. 1A). The Simple Plex assay workflow is summarized in Fig. 1c, consisting of eight main steps which are described in detail in the Methods section (Fig. 1C).

### Standard Curves

**Figure 2:**
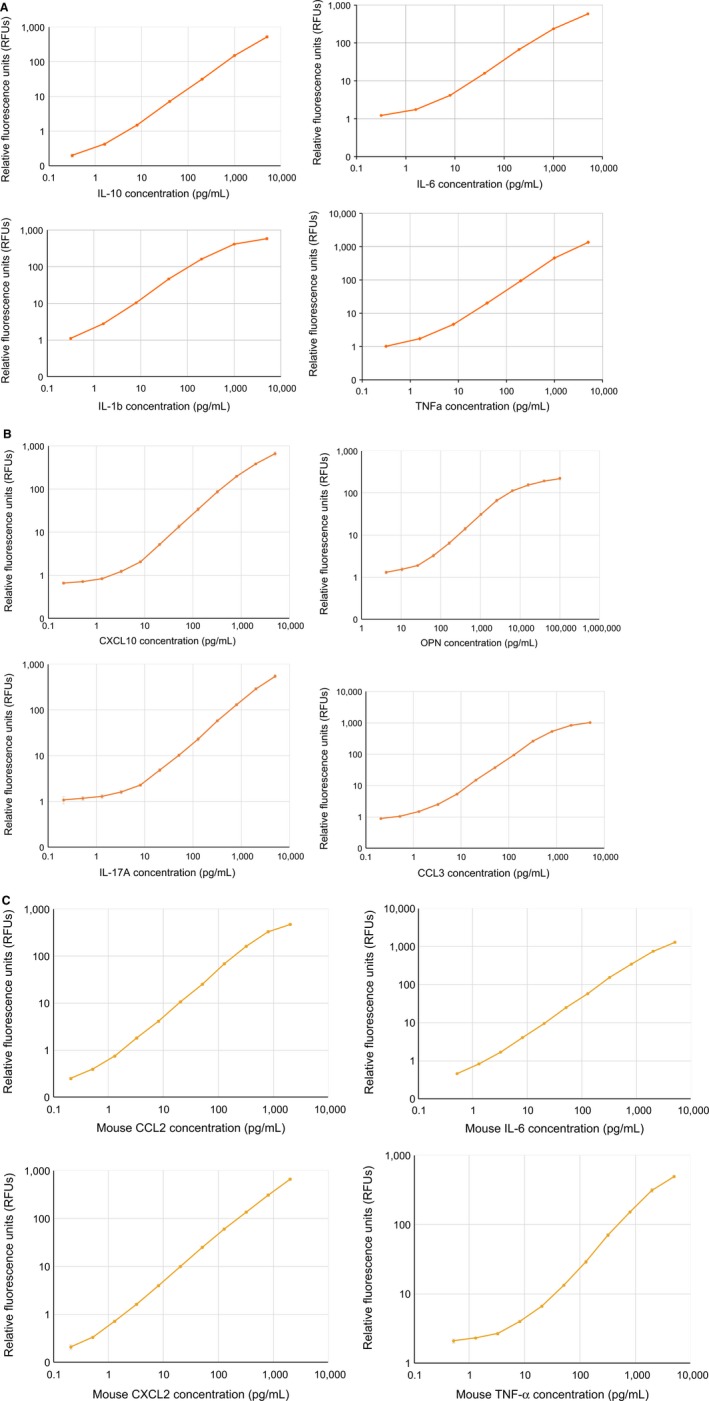
(A) Curves for Human Panel A: IL‐1β, IL‐6, IL‐10, and TNF‐α. (B) Curves for Human Panel B: CCL3, CXCL10; IL‐17, and OPN. (C) Curves for Mouse Panel: CCL2, CXCL2, IL‐6, and TNF‐α.

To determine linear ranges and sensitivity of the assay, specific capture and detection antibodies for eight human and four murine analytes were tested in panels of four (Human Panel A: IL‐1β, IL‐6, IL‐10, and TNF‐α; Human Panel B: IL‐17a, OPN, CXCL10, CCL3. Murine Panel: CCCL2, CXCL2, IL‐6, and TNF‐α). The upper limits of quantification (ULOQ), lower limit of quantification (LLOQ), and limit of detection (LOD) were established according to the FDA guidance as indicated in Table 1 legend and Methods. Standard eight or 13‐point curves (including zero) were generated by fivefold or 2.5‐fold serial dilution in calibration buffer (Fig. 2A for Human Panel A and Fig. 2B for Human Panel B). The standard curve for each biomarker showed dynamic ranges >2.8 to >3.5 order of magnitude with ULOQ ranges from 1000 to 6400 pg/mL, LLOQ ranges between 0.32‐8 pg/mL, and LOD ranges from 0.32 to 1.98 pg/mL for the eight human analytes (Table 1). Similarly, for the four murine analytes in the murine panel we established standard 13‐point curves (including zero) by 2.5‐fold serial dilution in calibration buffer (Fig 2C). The murine analytes also showed high sensitivity and broad linear ranges (Table 1). These LLOQ and ULOQ are sensitive enough to ensure that biomarker concentrations in multiple biological sample types can be detected and quantified.

### Effect of Multiplexing on Assay Sensitivity

**Figure 3:**
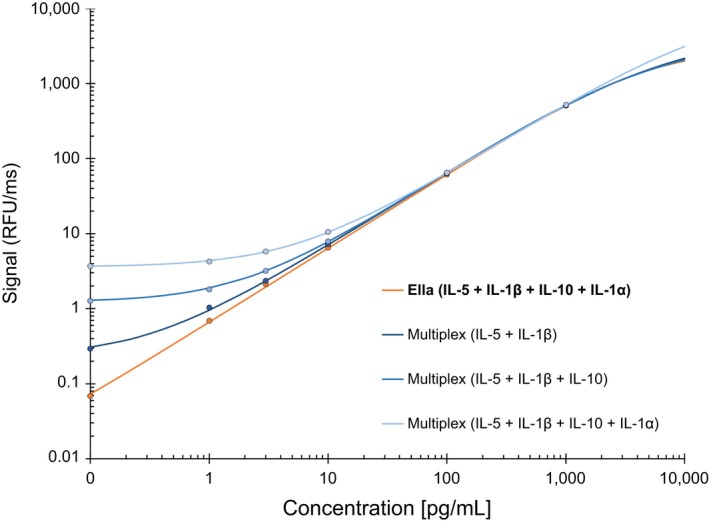
Effect of multiplexing antibodies to various analytes in the same GNR. The effect of cross‐reactivity was determined by adding increasing number of antibodies to the same GNR. Note that the addition of antibodies decreases the sensitivity of the assay.

One of the major problems when multiplexing is the loss of sensitivity due to cross‐reactivity between combined antibodies. Fig. 3 shows the advantage of the Simple Plex configuration, where each antibody is separated during the capture and detection steps allowing multi‐analyte detection without multiplexing. To evaluate the effect of multiplexing, we generated IL‐5 standard curves using Simple Plex format (one single capture antibody and one single detection antibody) compared with multiplex detection antibodies. The combination of additional detection antibodies into a multiplex cocktail decreased the sensitivity of the assay. Interestingly, we observed that each additional detecting antibody further decreased sensitivity for a single capture antibody (Fig. 3). These data demonstrate the benefit of multi‐analyte detection compared with multiplexing.

### Assay Reproducibility

**Figure 4:**
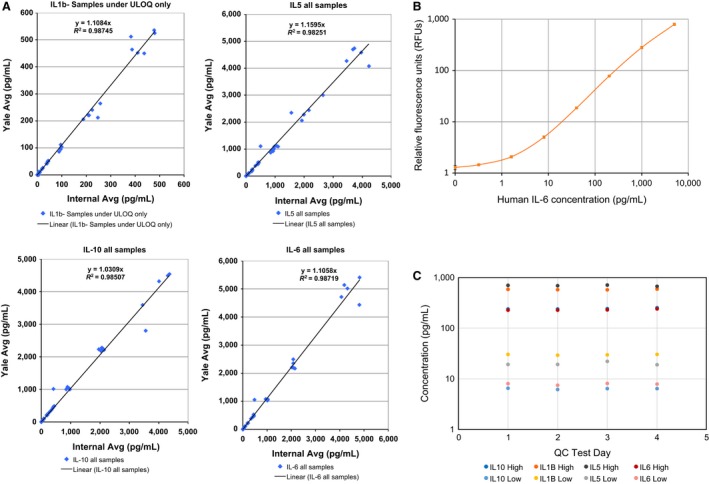
(A) Sample Reproducibility: Standard curve for analytes was generated by two separate laboratories at different times using the same batch of panels. Representative curves for four analytes. (B) Standard Curve Reproducibility: Stability and reproducibility of the assay was established by generating 11 individual standard curves for human IL‐6 run over the course of 31 days. (C) Intraassay Precision: Intraassay precision was determined for each analyte (IL‐10, IL‐1β, IL‐6, and IL‐5) by running cartridges containing eight replicates of either high or low QCs over a period of 4 days.

The stability and reproducibility of a platform is critical for the adequate evaluation of biosamples. Furthermore, the performance of the assay should not be affected by the source of biological sample (serum or plasma) or experimental variables. To examine the assay reproducibility of four analytes (IL1‐β, IL‐5, IL‐10, and IL‐6), 15 individual human samples (five normal human serum, five normal human plasma, two hemolyzed human plasma, and three lipemic human plasma) were run in duplicate inlets, each yielding triplicate GNR results for a total of six measurements. The same samples were run blind by two different users on two separate instruments and at two different locations. Each sample was run neat and spiked at low, medium, and high concentrations of the specific analytes. Data from the two sites were plotted, and linear correlations were determined for each analyte. As shown in Fig. 4A, all four analytes evaluated had *R* ^2^ value of 0.98 or higher, demonstrating an excellent concordance for measured values between the sites, instruments, and users for all the sample types tested.

### Stability and Reproducibility of the Simple Plex assay

Next, we evaluated the stability and reproducibility of the assay by generating 11 individual standard curves for human IL‐6 run over the course of 31 days. Each curve consisted of eight points (including zero) generated by fivefold serial dilutions in the calibration buffer. The generated curves were then backfit to factory‐generated calibration standard curves provided by the cartridge kit barcode. All 11 curves were consistent with each other and with factory calibration curves (Fig. 4B). The coefficient of variation (CV) for the relative fluorescence units (RFUs) at each concentration was 5% or less, suggesting a high level of precision and reproducibility (Table 2). When the 11 curves were backfit to the factory calibration curves, resultant calculated concentrations also demonstrated an excellent reproducibility. CV for calculated concentrations was less than 20% for all the curve points, suggesting all concentrations tested are within the limits of quantitation (Table 2)

**Table 2:** Stability and reproducibility of the Simple Plex assay

IL‐6 (pg/mL)	Count	Relative Fluorescence Units (RFUs)			Calculated Concentration (pg/mL)		
		Avg	StdDev	CV	Avg	StdDev	CV
0	11	1.29	0.12	10%			
0.32	11	1.45	0.07	5%	0.74	0.14	19%
1.6	11	2.08	0.10	5%	2.07	0.23	11%
8	11	5.02	0.27	5%	8.98	0.67	7%
40	11	18.61	0.72	4%	44.71	1.98	4%
200	11	77.84	2.41	3%	226.81	8.03	4%
1000	11	280.12	14.07	5%	1058.72	67.80	6%
5000	11	793.62	41.28	5%	5513.33	635.48	12%

### Assay Precision of the Simple Plex platform

To determine the intra‐ and interassay precisions for Simple Plex platform, we calculated the average low and high quality control (QC) values across multiple cartridges for four human analytes (IL‐10, IL‐1β, IL‐6, and IL‐5). We observed an excellent reproducibility for the low and high QCs between different cartridges run in different days (Fig. 4C). Furthermore, interassay precision was characterized by CVs ranging from 3 to 8% for both high and low QCs for all four tested analytes (Table 3).

**Table 3:** Assay precision of the Simple Plex platform

Average low QC interassay					Average high QC interassay			
Analyte	Count	Avg (pg/mL)	S.D	Backfit CV	Count	Avg (pg/mL)	S.D	Backfit CV
IL‐10	16	16	0.8	5%	24	797.3	22.9	3%
IL‐1b	16	17.7	0.6	3%	24	861	70.2	8%
IL‐6	16	15.7	0.8	5%	24	812.8	32.4	4%
IL‐5	16	17.6	1.5	8%	24	771.7	36.5	5%

Intraassay precision was determined for each analyte (IL‐10, IL‐1β, IL‐6, and IL‐5) by running cartridges containing eight replicates of either high or low QCs over a period of 4 days. Again, all four analytes showed an excellent intraassay precision, with CVs of 7% or less across both high and low QC concentrations, and across all QC replicate cartridges (Table 4).

**Table 4:** Intra‐assay precision of the Simple Plex platform

Average low QC intra‐assay					Average high QC intra‐assay			
Analyte	Count	Avg (pg/mL)	S.D	Backfit CV	Count	Avg (pg/mL)	S.D	Backfit CV
IL‐10	16	10.6	3.6	4%	24	793.92	17.66	2%
IL‐1b	16	15.9	2.3	3%	24	870.79	64.26	7%
IL‐6	16	15.3	0.4	3%	24	810.58	25.97	3%
IL‐5	16	16.1	1.5	5%	24	785.19	36.19	5%

### Efficacy of the Simple Plex Platform for the Analysis of Clinical Biological Samples

Our next objective was to determine the applicability of the platform for the quantitative analysis of different biological sample types by evaluating the concentrations of TNF‐α, a major pro‐inflammatory cytokine, in a variety of both human and murine sample types.

**Figure 5:**
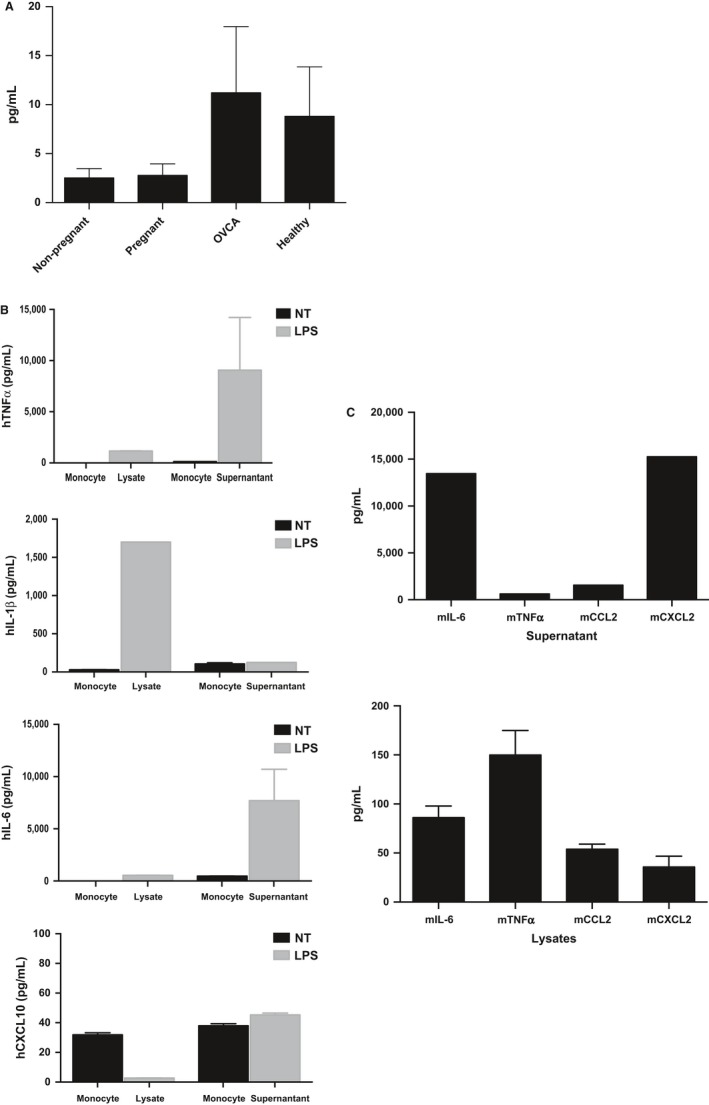
Efficacy of the Simple Plex Platform for the Analysis of Human and Murine Cellular Samples. (A) Detection of human cytokines and chemokines. Detection of circulating TNF‐α levels in serum samples revealing some differential expression levels between pre‐ and post‐menopausal women as well as between healthy post‐menopausal women and ovarian cancer patients. (B) Detection of cytokines and chemokines in peripheral blood monocytes stimulated with TLR4 ligand LPS. CD14 + peripheral monocytes were treated with or without LPS (10 ng/ml) for 24 hrs after which both protein expression and secretion were analyzed using the Simple Plex platform. Detection of TNF‐α, IL‐6, and IP‐10 (CXCL10) were all highly induced and secreted after LPS stimulation, while IL‐1β was only detected in the lysate. (C) Detection of murine cytokines and chemokines. Baseline peritoneal macrophage supernatant and spleen lysate were analyzed using the Simple Plex platform. Murine pro‐inflammatory cytokines TNF‐α, IL‐6, CCL2, and CXCL2 were all detected in the supernatant as well as cell lysate.

First, we looked at circulating TNF‐α concentration in serum obtained from several different female patients including the following: (i) non‐pregnant pre‐menopausal women (*n* = 7), (ii) pregnant women (*n* = 7), (iii) patients with ovarian cancer (*n* = 14), and (iv) healthy post‐menopausal women (*n* = 14). The Simple Plex platform was able to detect circulating TNF‐α levels in all the tested serum samples, revealing some differential expression levels between pre‐ and post‐menopausal women, as well as between healthy post‐menopausal women and ovarian cancer patients (Fig. 5A). These results suggest that Simple Plex sensitivity, combined with the platform's ease of use and minimal sample handling and small volume requirements, makes it an effective platform for the development of biomarker tests for clinical diagnosis.

### Efficacy of the Simple Plex Platform for the Analysis of Human Cellular Samples

Another important application of these technologies is the quantification of analytes for *in vitro* studies, especially in cellular supernatants and cell lysates. Consequently, we quantified pro‐inflammatory cytokine levels in human monocytes in the absence or presence of the Toll‐like receptor 4 (TLR‐4) ligand lipopolysaccharide (LPS). Our data show that LPS induces a significant increase in TNF‐α and IL‐6 intracellular expression (cell lysate) as well as secretion (supernatant) in peripheral blood monocytes (Fig. 5B). Interestingly, we observed a significant increase in IL‐1β only in the cell lysate but not in the supernatant (Fig. 5B), which is in accordance with previous published data suggesting the requirement for a second signal for the secretion of IL‐1β.^15^, ^16^ Interestingly, the intracellular IP‐10 (lysate) decreases following LPS treatment, whereas we observe a moderate increase in IP‐10 in the supernatant (Fig. 5B).

### Efficacy of the Simple Plex Platform for the Analysis of Murine Cellular Samples

We also evaluated the detection of pro‐inflammatory cytokines in representative immune organs and cells from mice. Spleen and peritoneal macrophages were collected from C57Bl/6 mice. Cell lysates were prepared from the spleen, and supernatants were collected from peritoneal macrophages after 48 hours of culture. Our data show that the Simple Plex platform was able to quantify the expression and secretion of IL‐6, TNF‐α, CCL2, and CXCL2 in all the tested samples (Fig. 5C).

### Simple Plex vs Luminex

**Figure 6:**
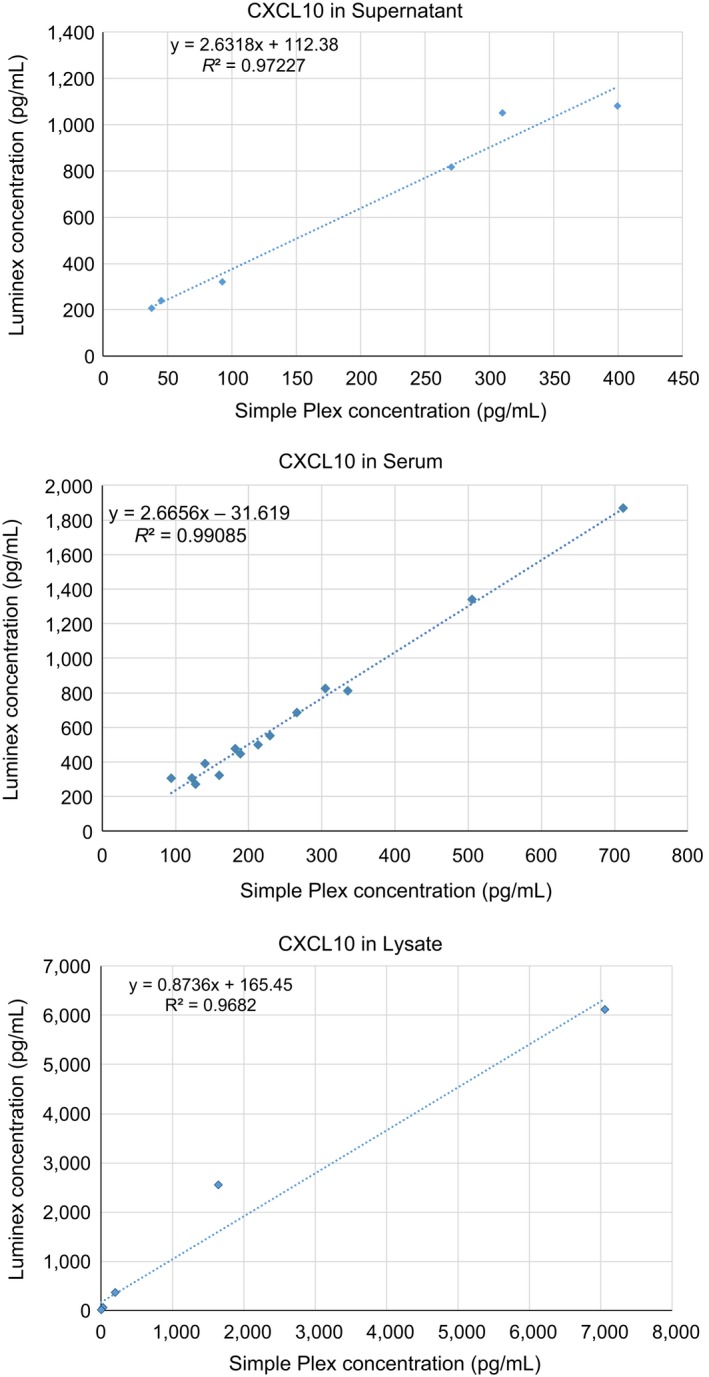
Correlation between the Luminex and Simple Plex platforms. Correlation of detection levels for CXCL10 in human biological samples between Luminex and Simple Plex platforms. CXCL10 expression levels showed a correlation *R* ^2^ of 0.97 or higher in all sample types.

One of the most widely used multiplex platforms is Luminex, which evaluates multiple analytes in bead‐based format where all the antibodies are combined in a single solution. Our next objective was to compare the quantification of analytes between the two platforms and determine their correlation. We performed both assays with multiple sample types (lysate, supernatant, serum) and assayed for four cytokines and chemokines (CXCL10, IL‐1β, IL‐6, and TNF‐α). In those samples and analytes that were in the range of Luminex sensitivity, we observed an excellent correlation between the two platforms. An example is shown in Fig. 6 for CXCL10, where all sample types have an *R* ^2^ of 0.97 or higher. Table 5 shows the correlation for the rest of the analytes evaluated (Table 5). However, certain low abundance analytes, such as TNF‐α in the serum and cell lysate, were detected by Simple Plex, but were below the levels of sensitivity for Luminex, thereby preventing correlation (Table 6).

**Table 5:** Correlation of Simple Plex vs Luminex

	Serum		Supernatant		Lysate	
Analyte	*n*	*R* ^2^	*n*	*R* ^2^	*n*	*R* ^2^
CXCL10	14	0.9908	6	0.9723	8	0.9682
IL‐1b	0	N/A	10	0.9902	5	0.9238
IL‐6	1	N/A	10	0.9310	8	0.9501
TNF‐a	2	N/A	3	0.9781	1	N/A

N/A = too many samples outside LOQs for one or both platforms.

**Table 6:** Detection sensitivity levels for Simple Plex and Luminex

Supernatant	TNFa (pg/mL)	
	Simple Plex	Luminex
Sample		
d2 LPS CM	OOR	OOR
d2 NT CM	128.88	4.72
R182 CM	6.63	OOR
Sw 1%	4.22	OOR
Sw LPS	1.98	OOR
Sw MHV	2.19	OOR
Sw NT	2.15	OOR
Sw polyIC	3.17	OOR
TEM LPS	625.03	85.36
TEM NT	33.38	4.49
TR182 CM	2.73	OOR
Lysate		
Sample		
d2 LPS lysate	1166.07	257.08
d2 NT lysate	1.99	OOR
R182 lysate	8.21	OOR
Sw LPS lysate	0.57	OOR
Sw MHV lysate	0.52	OOR
Sw NT lysate	0.77	OOR
Sw polyIC lysate	1.72	OOR
TEM LPS lysate	61.98	OOR
TEM NT lysate	7.21	OOR
TR182 lysate	1.99	OOR
Serum		
Sample		
NP1	4.17	105.66
NP2	10.77	OOR
NP3	5.90	OOR
NP4	4.55	OOR
NP5	8.72	OOR
NP6	5.57	OOR
NP7	12.33	OOR
P1	5.68	OOR
P2	4.85	OOR
P3	3.37	OOR
P4	5.30	OOR
P5	2.33	OOR
P6	4.52	OOR
P7	9.14	OOR

### Simple Plex vs ELISA

**Figure 7:**
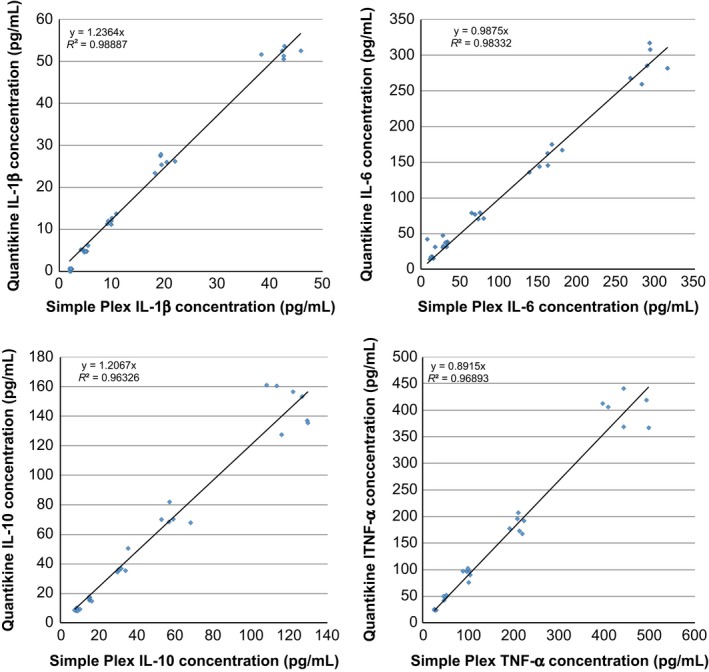
Correlation between the ELISA and Simple Plex platforms. Correlation of detection levels for CXCL10, IL‐1β, IL‐6, IL‐10, and TNF‐α in human biological samples between ELISA and Simple Plex platforms. All the tested cytokine expression levels showed a correlation *R* ^2^ of 0.96 or higher in all sample types.

The gold standard immunoassay for the quantification of cytokines and chemokines is the enzyme‐linked immunosorbent assay (ELISA). Our final objective was to compare the quantification of analytes between the two platforms and determine their correlation. We ran blind 32 identical human plasma and serum samples per analyte and plotted Simple Plex‐measured concentration (pg/ml) against Quantikine‐measured concentration (pg/ml) to determine the linear correlation. All the evaluated analytes had an R^2^ value of 0.96 or higher (Fig. 7). To cover the entire dynamic range of both assays, in certain cases serum or plasma samples were spiked with human cells supernatant containing elevated levels of endogenous analytes.

### Comparison of Dynamic Ranges of Tested Immunoassay Platforms

**Figure 8:**
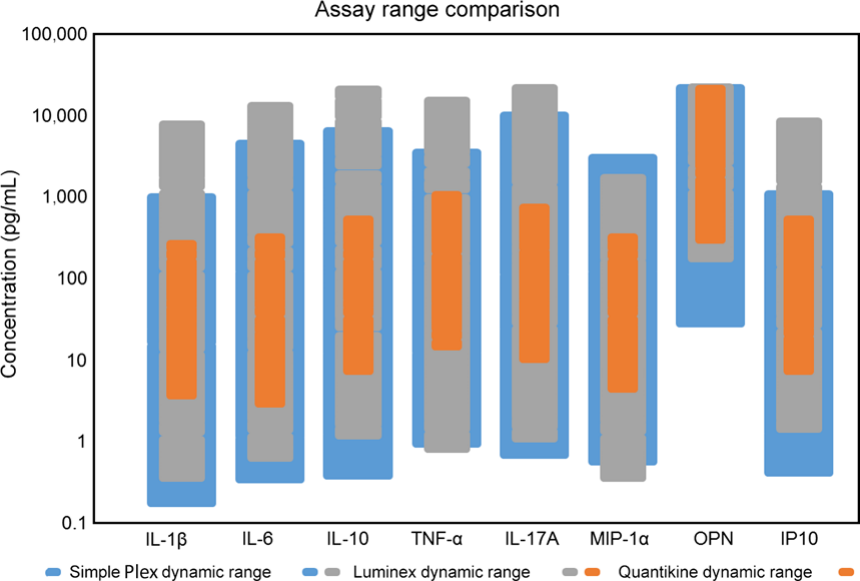
Dynamic Ranges of All Three Tested Immunoassay Platforms. Limits of quantitation (LLOQ and ULOQ) were obtained from current manufacturer‐provided specification sheets or product datasheets for all eight human analytes evaluated. Each bar for each analyte represents the platform's dynamic range for that assay, as defined by reported LLOQ and ULOQ values.

To directly compare the relative dynamic ranges across all three immunoassay platforms, limits of quantitation (LLOQ and ULOQ) were obtained from current manufacturer‐provided specification sheets or product datasheets for all 8 human analytes evaluated. These data are presented in both graphical (Fig. 8) and table formats (Table 7). Compared with Luminex, Simple Plex assays were more sensitive for six of eight analytes and overall dynamic ranges were comparable. Compared with Quantikine ELISA, Simple Plex assays demonstrated superior low‐end sensitivity and broader dynamic range for all eight assays.

**Table 7:** Dynamic ranges of tested immunoassay platforms

Analyte	Simple Plex		BioPlex Chemokine		Quantikine	
	LLOQ (pg/mL)	ULOQ (pg/mL)	LLOQ (pg/mL)	ULOQ (pg/mL)	LLOQ (pg/mL)	ULOQ (pg/mL)
IL‐1b	0.21	840	0.4	7000	3.9	250
IL‐6	0.41	3850	0.7	12000	3.13	300
IL‐10	0.46	5530	1.3	18708	7.8	500
TNFa	1.14	2990	0.9	13879	15.6	1000
IL‐17A	0.82	8490	1.2	19682	10.9	700
MIP‐1a	0.68	2550	0.4	1543	4.7	300
OPN	34.3	18545	195	20000	312	20000
IP10	0.49	920	1.6	7714	7.8	500

## Conclusion

We describe the characterization and validation of Simple Plex, a novel multi‐analyte, automated microfluidic platform that allows the evaluation of cytokines and chemokines from human and mice biological samples. Our data demonstrate an excellent correlation between the results obtained with Simple Plex and conventional immunoassays such as ELISA and Luminex. However, Simple Plex showed major advantages over these traditional plate‐based immunoassay approaches for multiplexing in terms of required sample volumes, high sensitivity and dynamic range, coefficient of variation, and reproducibility.

## Supporting information


**Figure S1.** Components of the Simple Plex immunoassay system: The desktop Ella Analyzer.
**Figure S2.** Components of the Simple Plex immunoassay system: The Simple Plex microfluidic cartridge.
