# Supplementary material for: Simple Plex™: A Novel Multi‐Analyte, Automated Microfluidic Immunoassay Platform for the Detection of Human and Mouse Cytokines and Chemokines
Source: Am J Reprod Immunol. 2016 May 11;75(6):678–93. doi: 10.1111/aji.12512 (PMC5084752; doi:10.1111/aji.12512)
Supplement: Supplementary file 1 — Figure S1. Components of the Simple Plex immunoassay system: The desktop Ella Analyzer. Figure S2. Components of the Simple Plex immunoassay system: The Simple Plex microfluidic cartridge. [file AJI-75-678-s001.pdf]

Supplemental Figure 1

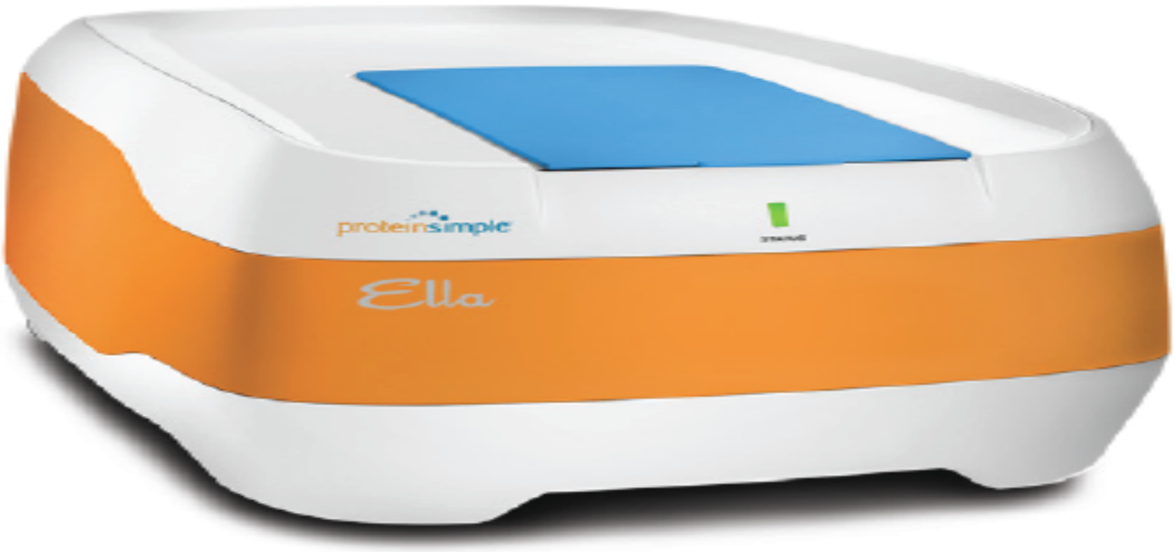

Supplemental Figure 2

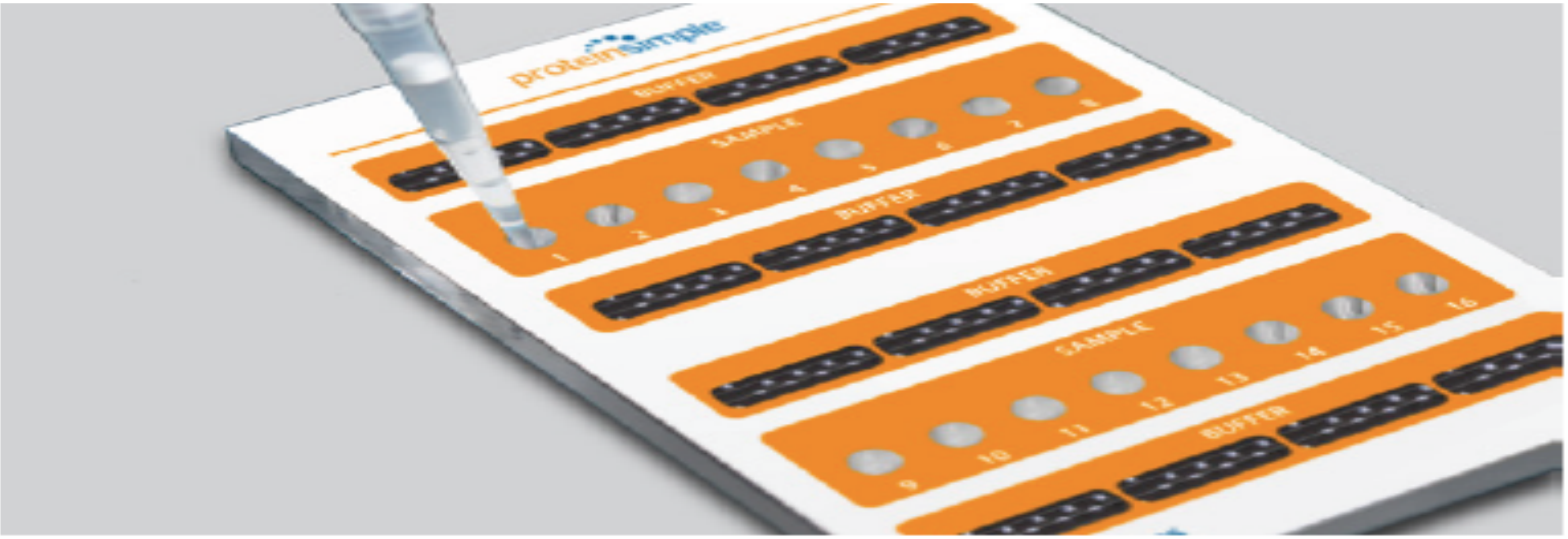

Supplementary Figure Legends

Sup 1 and 2. Components of the Simple Plex immunoassay system:

1) **The desktop Ella Analyzer**, a microfluidic analyzer connected to a PC computer via USB and Ethernet cables. The PC has the Simple Plex Runner instrument control software, the Simple Plex Explorer data analysis software, and a barcode scanner to streamline data entry.

2) **The Simple Plex microfluidic cartridge**, an assay kit that contains a disposable cartridge, and all the reagents required to run the test.
